# Primary cCT Imaging Based Clinico-Neurological Assessment—Calling for Addition of Telestroke Video Consultation in Patients With Intracerebral Hemorrhage

**DOI:** 10.3389/fneur.2018.00607

**Published:** 2018-07-26

**Authors:** Andrea Wagner, Karl-Michael Schebesch, Florian Zeman, Stefan Isenmann, Andreas Steinbrecher, Thomas Kapapa, Dobri Baldaranov, Roland Backhaus, Felix Schlachetzki

**Affiliations:** ^1^Department of Neurology, University Regensburg, Regensburg, Germany; ^2^Department of Neurosurgery, University Medical Center Regensburg, Regensburg, Germany; ^3^Center for Clinical Studies, University Medical Center Regensburg, Regensburg, Germany; ^4^Department of Neurology, HELIOS University Hospital Wuppertal—University Witten/Herdecke, Wuppertal, Germany; ^5^Department of Neurology, General Hospital HELIOS Klinikum Erfurt, Erfurt, Germany; ^6^Department of Neurosurgery, University Medical Center Ulm, Ulm, Germany; ^7^Department of Neurology, University Regensburg, Regensburg, Germany; ^8^Stroke Center Hirslanden, Klinik Hirslanden Zurich, Zurich, Switzerland; ^9^Department of Neurology, University Regensburg, Regensburg, Germany

**Keywords:** intracerebral hemorrhage, quality of life, outcome, computed tomography, telestroke

## Abstract

**Background and Purpose:** Intracerebral hemorrhage (ICH) requires rapid decision making to decrease morbidity and mortality although time frame and optimal therapy are still ill defined. Ideally, specialized neurologists, neurosurgeons, and (neuro-) radiologists who know the patient's clinical status and their cerebral computed tomography imaging (cCT) make a joint decision on the clinical management. However, in telestroke networks, a shift toward cCT imaging criteria used for decision making can be observed for practical reasons. Here we investigated the “reverse correlation” from cCT imaging to the actual clinical presentation as evaluated by the Glasgow Coma Scale (GCS) and the National Institutes of Health Stroke Scale (NIHSS).

**Methods:** CCT images and basic information (age, sex, and time of onset) of 50 patients with hypertensive and lobar ICH were presented to 14 experienced neurologists and 15 neurosurgeons. Based on this information, the NIHSS and GCS scores were estimated for each patient. The differences between the actual GCS and NIHSS scores and the cCT-imaging-based estimated scores were plotted in a bland-Altman plot.

**Results:** The average estimated GCS score mainly based on cCT imaging was 12. 4 ± 2.8 (actual value: 13.0 ± 2.5; *p* = 0.100), the estimated NIHSS score was 13.9 ± 9.1 (actual value: 10.8 ± 7.3; *p* < 0.001). Thus, in cCT-imaging-based evaluation, the neurological status of patients especially employing the NIHSS was estimated poorer, particularly in patients with lobar ICH. “Reverse clinical” evaluation based on cCT-imaging alone may increase the rate of intubation and secondary transferal and neurosurgical treatment. Telestroke networks should consider both, videoassessment of the actual clinical picture and cCT-imaging findings to make appropriate acute treatment decisions.

## Introduction

Spontaneous intracerebral hemorrhage (ICH) accounts for 10 to 15% of all strokes in Western populations, and the case fatality ranges between 40 and 55% (1). In patients with acute ICH, physicians need to immediately decide whether surgical or conservative treatment might be the best option for the patient. Often, patients are initially seen in primary care hospitals lacking specific neurological or neurosurgical departments. Non-specialized neurological examinations are increasingly followed by reading a patient's cerebral computed tomography imaging(cCT) via teleradiology consultation. In the case of ischemic stroke, the decision to conduct thrombolysis or even endovascular treatment basically relies on the NIHSS score, a cCT scan excluding ICH, an appropriate time window and, the status of the arteries leading to the brain (2). The NIHSS was primarily designed for estimation of the severity of clinical symptoms in patients suitable for thrombolysis (3), has few items, is quickly to perform and has excellent interrater reliability, supported by available online certification(4). In acute stroke therapy telemedicine is established in Germany in networks like the TeleMedical Project for integrative Stroke Care (TEMPiS) in Bavaria, Germany, since 15 years now and has significantly increased the rate of treated strokes and transient ischemic attacks as well as decreased the onset-to-treatment and door-to-needle time in clinically underserved areas (5). For clinical decision making, e.g., in thrombolysis, teleradiology using electronically transmitted original imaging data has to be completed by teleconsultation by a remotely located expert through the use of high quality videoconferencing (6). The same concept could also be used for the first neurological consultation of patients with intracerebral hemorrhage in rural areas.

However therapeutic strategies in ICH are less clearly defined, and clinical presentation may vary considerably depending on the ICH-specific information gained by the cCT scan. Significant predictive factors are the volume of intraparenchymal blood on the initial cCT scan, presence, and ongoing expansion of intraventricular hemorrhage (IVH), and the location and expansion of hematoma (7). The ICH score is the most widely used outcome score in this context, reflecting the level of consciousness, age, ICH volume, IVH, and the infratentorial location of ICH. These parameters are independent predictors for both acute and long-term outcome (7–9). Because patients with lobar ICH have a different clinical background than patients with non-lobar ICH, the outcome may also differ (1).

To the best of our knowledge, the accuracy of estimating the initial cliniconeurological presentation of a patient by means of a cCT scan, here termed “reverse correlation,” has not been investigated in a study. Therefore, our objective was to evaluate how precisely neurologists and neurosurgeons are able to estimate a patient's clinical status when presented with the patient's first cCT scan but without or with only minimal information on the clinical status of the patient. This is of importance for decision making with growing stroke networks primarily relying on (neuro)radiological consultation.

## Materials and methods

The anonymized files of the initial diagnostic cCT scans of 50 patients with acute neurological deficit and acute ICH on a CD-Rom were send to 20 board-certified neurologists and 20 board-certified neurosurgeons. Of these, 25 patients had signs of acute symptomatic deep ICH [also classified as hypertensive (deep) “typical” ICH due to the location, i.e. the basal ganglia, pons, or cerebellum (10)] and 25 patients had lobar “atypical” ICH. The raters were also provided only with additional information on patient age, sex, and the time between symptom onset and cCT imaging (see Table 1). As the patient data were only used in anonymized form to present them to the physicians neither local ethic approval nor patient consent was necessary. Patients were excluded if they had other causes of ICH such as excessive use of a vitamin K antagonist (INR >3), antecedent head trauma or ischemic stroke, CNS tumor, vascular malformation, vasculitis, blood dyscrasia, or coagulopathy (11). Patients with lobar ICH were only included if they had possible or probable cerebral amyloid angiopathy (CAA) according to the modified Boston criteria (12). These patients routinely underwent MRI with MR-angiography or CT-angiography to rule out any vasculopathy and to detect microbleedings or lobar bleeds in cortical regions or cortical superficial siderosis, which defines probable CAA in the later course of the disease (12). Median age of the patients was 71 years (range: 39 to 97 years) and 26/50 were women. Time from symptom onset to initial cCT-imaging was less than 1.5 h in 12 patients, 1.5 h to 4.5 h in 11 patients, 4.5 h to 12 h in 11 patients, and more than 12 h in 16 patients. This information was given to the raters.

**Table 1:** Patients characteristics: age, sex, time between symptom onset and initial cCT scan, actual GCS and NIHSS scores, medium GCS and NIHSS scores estimated by the neurologists or neurosurgeons on basis of the presented cCT scan and the basic information about the patient, delta GCS and NIHSS scores as the difference between the actual and the estimated GCS and NIHSS scores.

**Pat**	**Gen-der**	**Age (years)**	**Type of ICH**	**Time between symptom onset and initial cCT**	**Actual NIHSS**	**Medium estimated NIHSS**	**Δ NIHSS**	**Actual GCS**	**Medium estimated GCS**	**Δ GCS**
1	F	76	Atypical	>4.5 and <12 h	20	17.71	2.29	12	9.69	2.31
2	M	63	Typical	>12 h	13	16.60	−3.60	12	9.83	2.17
3	F	84	Atypical	>4.5 and <12 h	6	5.30	0.70	15	13.52	1.48
4	M	70	Atypical	>12 h	2	3.90	−1.90	15	14.21	0.79
5	F	76	Atypical	<1.5 h	10	3.20	6.80	12	13.93	−1.93
6	M	65	Typical	>12 h	19	19.55	−0.55	14	8.45	5.55
7	F	80	Atypical	>12 h	3	4.00	−1.00	15	14.10	0.90
8	M	75	Typical	<1.5 h	19	13.35	5.65	10	11.83	−1.83
9	F	57	Typical	>1.5 and <4.5 h	21	19.50	1.50	8	8.97	−0.97
10	F	71	Typical	>12 h	4	7.95	−3.95	15	13.96	1.04
11	M	43	Typical	>12 h	15	11.90	3.10	15	12.90	2.10
12	F	73	Atypical	<1.5 h	12	7.16	4.84	15	12.85	2.15
13	F	89	Typical	>12 h	1	6.15	−5.15	13	14.00	−1.00
14	F	84	Atypical	>12 h	7	11.45	−4.45	11	12.07	−1.07
15	M	82	Atypical	>4.5 and <12 h	4	6.60	−2.60	14	13.62	0.38
16	M	61	Atypical	<1.5 h	4	4.80	−0.80	15	13.97	1.03
17	M	56	Atypical	<1.5 h	4	12.70	−8.70	11	11.69	−0.69
18	F	83	Atypical	>12 h	24	14.10	9.90	11	11.52	−0.52
19	F	70	Atypical	>4.5 and <12 h	13	13.30	−0.30	15	11.14	3.86
20	F	91	Typical	>1.5 and <4.5 h	12	11.68	0.32	10	12.41	−2.41
21	M	72	Atypical	>1.5 and <4.5 h	3	3.20	−0.20	15	14.52	0.48
22	F	97	Atypical	>1.5 and <4.5 h	20	25.90	−5.90	7	5.52	1.48
23	F	92	Typical	>1.5 and <4.5 h	7	15.45	−8.45	14	10.90	3.10
24	M	68	Atypical	>1.5 and <4.5 h	16	20.85	−4.85	10	7.45	2.55
25	F	87	Typical	>1.5 and <4.5 h	16	18.05	−2.05	15	10.79	4.21
26	F	81	Atypical	<1.5 h	4	15.75	−11.75	13	10.96	2.04
27	M	76	Typical	<1.5 h	22	22.53	−0.53	8	7.78	0.22
28	M	68	Atypical	<1.5 h	7	6.21	0.79	15	14.11	0.89
29	F	69	Atypical	>12 h	3	5.70	−2.70	15	13.96	1.04
30	F	62	Atypical	>4.5 and <12 h	3	7.35	−4.35	15	13.82	1.18
31	M	74	Atypical	>1.5 and <4.5 h	11	13.00	−2.00	15	11.54	3.46
32	F	51	Typical	<1.5 h	11	10.42	0.58	15	13.70	1.30
33	M	72	Typical	<1.5 h	23	22.10	0.90	8	9.07	−1.07
34	F	76	Atypical	>4.5 and <12 h	4	8.80	−4.80	13	13.68	−0.68
35	F	39	Typical	>1.5 and <4.5 h	25	22.55	2.45	8	7.75	0.25
36	M	85	Typical	>4.5 and <12 h	8	11.15	−3.15	15	13.43	1.57
37	M	66	Typical	<1.5 h	14	20.85	−6.85	13	9.14	3.86
38	M	53	Typical	>4.5 and <12 h	9	6.60	2.40	15	14.36	0.64
39	F	67	typical	<1.5 h	13	16.45	−3.45	15	9.86	5.14
40	M	90	Typical	>4.5 and <12 h	27	20.45	6.55	7	7.64	−0.64
41	M	64	Typical	>4.5 and <12 h	11	11.20	−0.20	14	13.29	0.71
42	M	75	Typical	>12 h	4	10.10	−6.10	14	13.07	0.93
43	M	52	Typical	>12 h	3	8.25	−5.25	15	13.68	1.32
44	F	74	Atypical	>12 h	2	7.40	−5.40	13	13.57	−0.57
45	F	77	Typical	>1.5 and <4.5 h	8	9.95	−1.95	15	13.86	1.14
46	M	77	Atypical	>12 h	15	8.00	7.00	14	12.70	1.30
47	M	59	Typical	>12 h	12	15.50	−3.50	14	11.39	2.61
48	F	66	Atypical	>12 h	3	3.70	−0.70	15	14.39	0.61
49	M	63	atypical	>4.5 and <12 h	2	11.65	−9.65	15	11.21	3.79
50	F	55	Typical	>1.5 and <4.5 h	22	13.10	8.90	10	12.64	−2.64

*F, female, M, male; ICH, intracerebral hemorrhage; cCT, cerebral computed tomography; NIHSS, National institute of health stroke scale; GCS, Glasgow coma scale*.

All raters were asked to estimate the NIHSS and GCS scores according to the cCT scan and the aforementioned basic clinical information. These data were compared with the GCS and NIHSS scores calculated on the basis of the patients' medical records at admission (here called “actual GCS/NIHSS”). Such comparison has been shown to be an appropriate method in previous studies (13). However, previous papers only could prove that for ischemic stroke, but considering the clinical parallels between ischemic and hemorrhagic stroke, we think it is adequate to also use this score for ICH.

Agreement between the actual and estimated NIHSS and GCS scores was evaluated on an individual patient basis, i.e., the patient's actual value was compared with one estimated value and calculated as the mean value of the estimate by all neurologists and neurosurgeons. The extent of agreement between the estimated and the actual NIHSS and GCS scores was quantified with the Bland-Altman plot (3). According to Krouwer (14), the actual NIHSS or GCS values rather than the average value were used on the X-axis. All plots include the mean difference, the 95% limits of confidence, and the regression line. Mean scores between the actual and estimated values were compared with a paired *t*-test. Differences between deep ICH and lobar ICH were analyzed with the Student's *t*-test. A *p*-value < 0.05 was considered statistically significant. All analyses were performed using R (version 3.3.3, The R Foundation for Statistical Computing).

## Results

Fourteen of Twenty neurologists and Fifteen of Twenty neurosurgeons addressed answered our questionnaire. The actual mean GCS score of the total ICH patient collective was 14 [standard deviation (SD) 2.5; range 7–15], and the actual mean NIHSS score was 10 (SD 7.3; range 1 to 27) (Table 1). Plotting the difference between the actual GCS and NIHSS scores and the imaging-based estimated scores, we found that—on average—the GCS score was estimated lower than the actual GCS score which was not statistically significant (actual value: 13.0 ± 2.5 vs. estimated value: 12.4 ± 2.8, *p* = 0.100). In contrast, the NIHSS score was—on average—statistically significant estimated higher than the actual NIHSS score (actual value: 10.8 ± 7.3 vs. estimated value: 13.9 ± 9.1, *p* < 0.001). Thus, the raters had overestimated the patients' neurological deficits.

**Figure 1:**
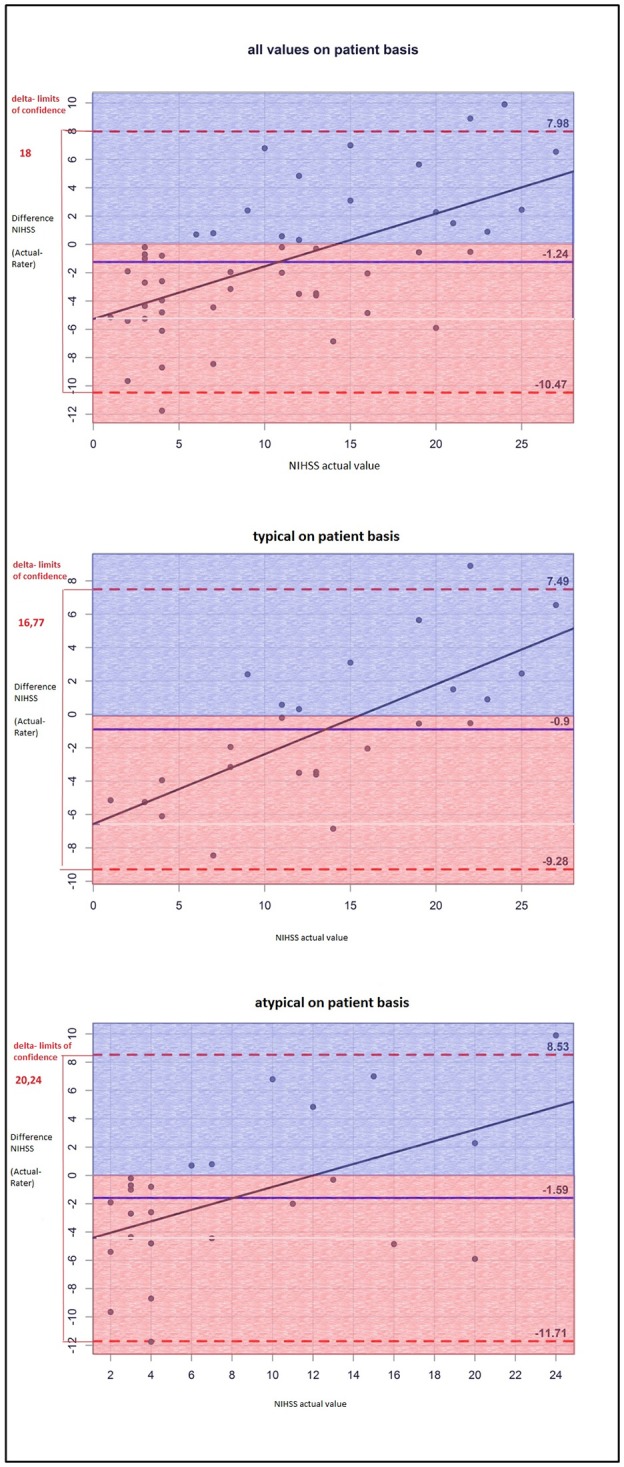
The Bland-Altman-plot shows the difference between the actual and the estimated NIHSS scores (mean for all raters) on a patient basis: The mean estimated NIHSS score is too high by 1.24. For deep ICH, the delta of limits of confidence is smaller, which means the differences between the rated and the actual NIHSS scores are smaller; thus, the NIHSS score is estimated more accurately than that of lobar ICH.

**Figure 2:**
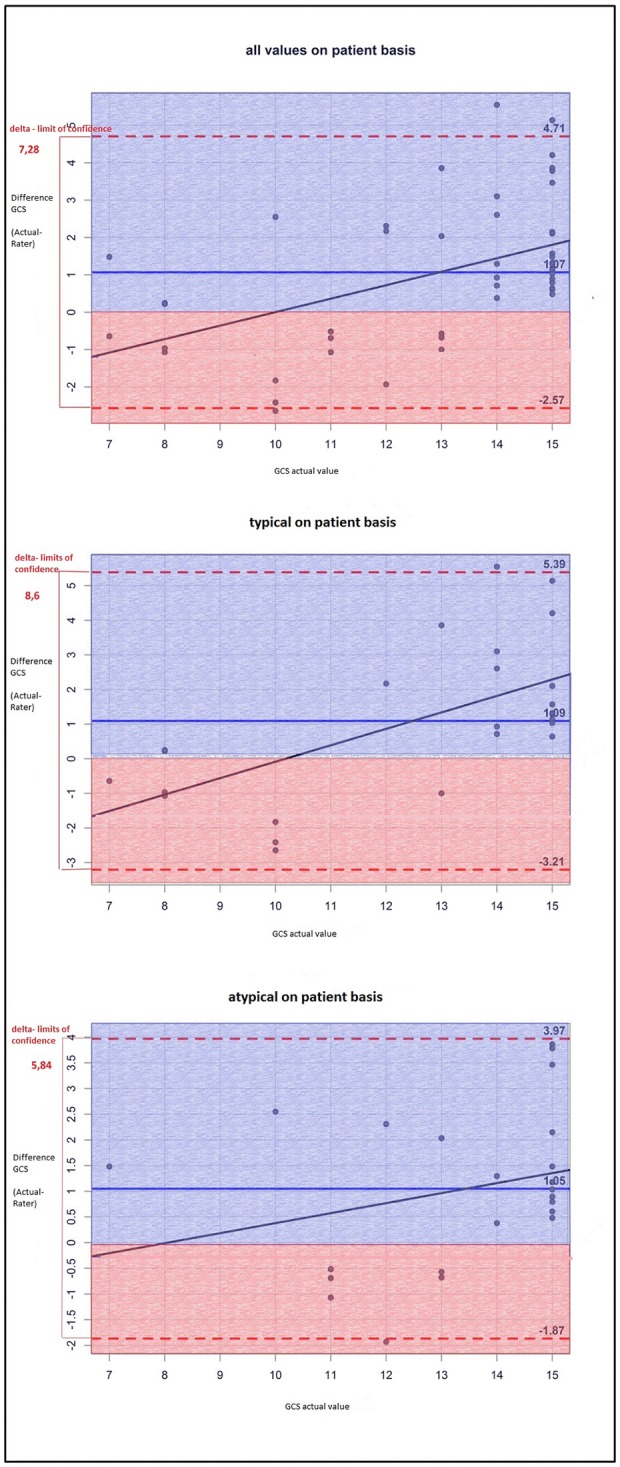
The Bland-Altman-plot shows the difference between the actual and the estimated GCS scores (mean for all raters) on patient basis: On average, the estimated GSC score is too low by 1.07 without any significant differences between typical (i.e., deep) and atypical (i.e., lobar) ICH.

The NIHSS score estimated on the basis of the cCT-imaging findings correlated better with the actual values in patients with hypertensive ICH than in patients with lobar ICH (Figure 1). The same finding applied to GCS scores (Figure 2), but the results were not statistically significant: The difference between the actual and the rated GCS values was −0.80 for atypical ICH (95%–CI: −1.80, 0.20) vs. a difference of −0.28 for typical ICH (95%–CI: −1.16, 0.60), *p* = 0.42. The difference between the actual and the rated NIHSS values was larger for atypical ICH (3.20) (95%–CI: 0.60, 5.80) than for typical ICH (2.92) (95%–CI: 0.60, 5.24), *p* = 0.869.

**Figure 3:**
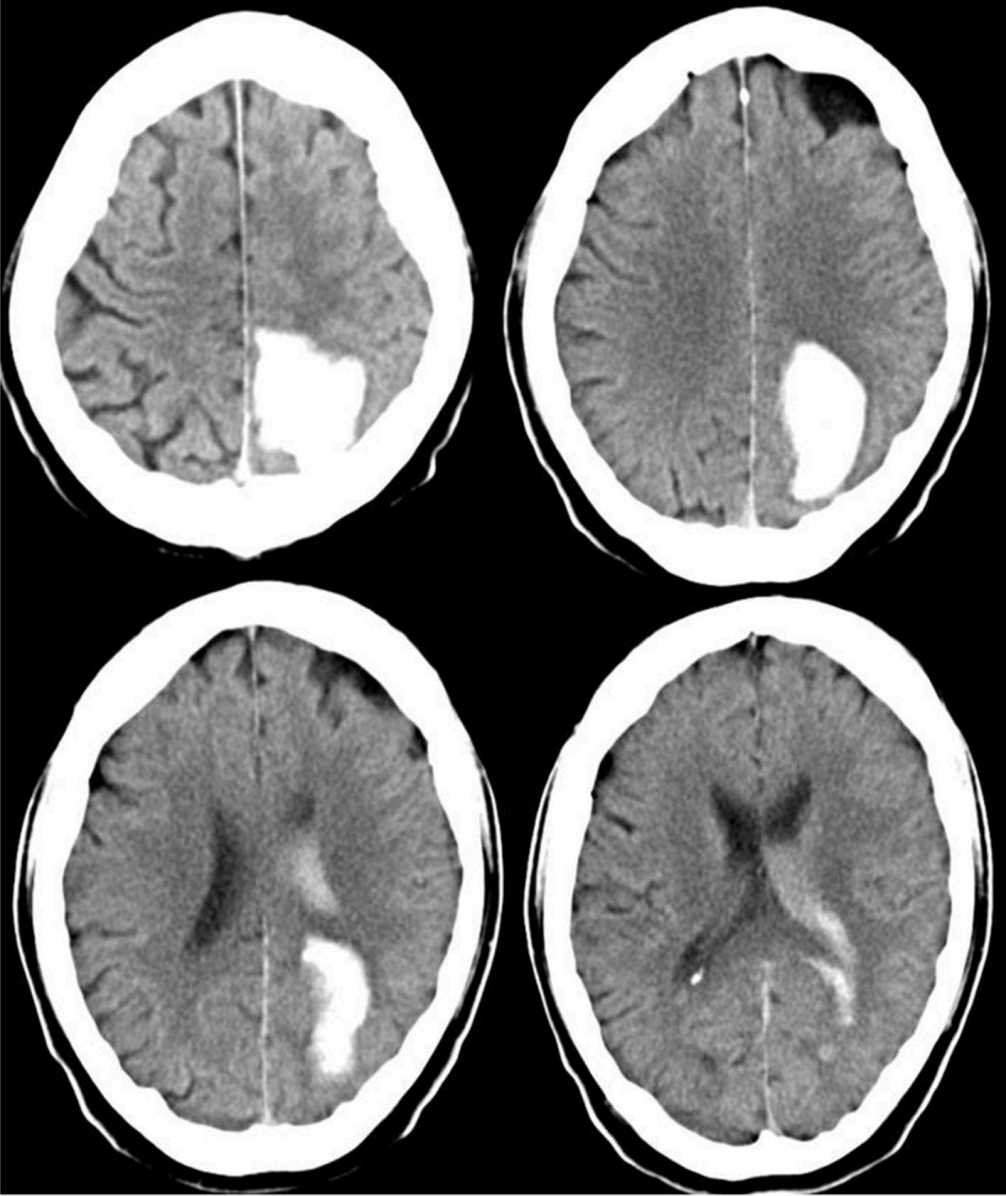
cCT scan of a 63-year old man, acquired between 4.5 and 12 h after symptom onset, showing left occipital ICH. Hemianopia to the left side resulted in a NIHSS score of 2 and in a GCS score of 15. The mean estimated NIHSS score was 11.2, the mean estimated GCS score 11.65.

Therefore, the raters had fewer problems to properly appraise the actual clinical status of patients with deep ICH than that of patients with lobar ICH. Particularly patients with a low actual NIHSS score were often given higher scores by the raters, whereas patients with a high actual NIHSS score were given lower scores as you can see in the single patient analysis shown as dots in the Bland-Altman plots (Figures 1, 2). The large confidence intervals of NIHSS and GCS score estimates both for the total patient population and the subgroups with deep and lobar ICH showed wide variation between the raters' answers. A typical cCT scan of a patient resulting in overestimation of the clinical presentation by the raters is shown in Figure 3.

## Discussion

In this study, we investigated the “reverse correlation” of experienced neurologists and neurosurgeons examining the cCT scans of patients with ICH, who estimated GCS and NIHSS scores on the basis of only basic additional information on age, sex, and time from symptom onset to imaging. Our study shows that even board-certified neurologists with years of experience in supervising a stroke unit or a neurological ICU or neurosurgeons who regularly treat patients with ICH (see Tables 2, 3) encounter considerable difficulties in estimating the correct scores, which may lead to over-rating the clinical symptoms of patients. In fact, the extent to which estimated and actual scores differed was rather sobering. We also observed a tendency to avoid both high as well as low extremes of the NIHSS and GCS scores. This study is highly relevant in times of growing specialization in medicine and of expanding telemedicine networks.

**Table 2:** Age and years of experience of board-certified neurologists and neurosurgeons-raw data.

**Physician**	**Group of physician**	**Age**	**Years of practice after becoming board certified neurologist/neurosurgeon**	**Years of leading a stroke unit (neurologists only)/ number of operated ICH as board certified neurosurgeon (neurosurgeons only)**
Neurologist 1	Neurologist	55	22	0
Neurologist 2	Neurologist	45	12	6
Neurologist 3	Neurologist	38	5	2
Neurologist 4	Neurologist	40	5	1
Neurologist 5	Neurologist	51	16	9
Neurologist 6	Neurologist	39	5	1
Neurologist 7	Neurologist	48	15	8
Neurologist 8	Neurologist	52	16	10
Neurologist 9	Neurologist	44	10	10
Neurologist 10	Neurologist	52	16	12
Neurologist 11	Neurologist	46	10	0
Neurologist 12	Neurologist	44	8	3
Neurologist 13	Neurologist	41	5	4
Neurologist 14	Neurologist	48	12	0
Neurosurgeon from own hospital 1	Neurosurgeon from own hospital	n.a.	n.a.	n.a.
Neurosurgeon from own hospital 2	Neurosurgeon from own hospital	n.a.	n.a.	n.a.
Neurosurgeon from own hospital 3	Neurosurgeon from own hospital	37	1	30
Neurosurgeon from own hospital 4	Neurosurgeon from own hospital	40	5	150
Neurosurgeon from own hospital 5	Neurosurgeon from own hospital	60	26	100
Neurosurgeon from own hospital 6	Neurosurgeon from own hospital	36	1.5	50
Neurosurgeon from own hospital 7	Neurosurgeon from own hospital	35	1	60
Neurosurgeon from own hospital 8	Neurosurgeon from own hospital	42	9	200
Neurosurgeon from own hospital 9	Neurosurgeon from own hospital	50	20	100
Neurosurgeon from own hospital 10	Neurosurgeon from own hospital	52	17	200
Neurosurgeon from other hospital 1	Neurosurgeon from other hospital	41	5	50
Neurosurgeon from other hospital 2	Neurosurgeon from other hospital	42	10	70
Neurosurgeon from other hospital 3	Neurosurgeon from other hospital	35	3	30
Neurosurgeon from other hospital 4	Neurosurgeon from other hospital	39	7	50
Neurosurgeon from other hospital 5	Neurosurgeon from other hospital	36	5	20

*n.a., not available*.

**Table 3:** Age and years of experience of board-certified neurologists and neurosurgeons and years of supervision of a stroke unit (neurologists only) or the number of operated ICH (neurosurgeons only)–statistics.

	**All (number)**	**Neurosurgeon (number)**	**Neurologist (number)**
**AGE OF PHYSICIAN (YEARS)**			
<40	8	6	2
40–<50	12	4	8
≥50	7	3	4
n.a.	2	2	0
**YEARS OF PRACTICE AFTER BECOMING BOARD CERTIFIED**			
**NEUROLOGIST/NEUROSURGEON**			
<5	4	4	0
5–<10	10	5	5
>10	13	4	9
n.a.	2	2	0
**YEARS OF LEADING A STROKE UNIT (NEUROLOGISTS ONLY)**			
<5			8
>5			6
**NUMBER OF OPERATED ICH (NEUROSURGEONS ONLY)**			
<100		8	
>100		5	
n.a.		2	

For patients with ICH, deep location and mass effects as well as some laboratory and clinical parameters [scored here by CSS (Canadian stroke scale), a score combining the level of consciousness, the degree of limb paresis and communication abnormalities] as well as systolic blood pressure at presentation were factors probably associated with poor outcome (15, 16).

Further relevant predictors for outcome and functional independence after 100 days are age and the NIHSS score at initial presentation, regardless of the location of the ICH (17, 18). In addition to the ICH score, the GCS score is also the most important independent predictor for patient outcome (9). Our study showed that solely relying on a cCT scan for clinical decision-making would lead to erroneous over-estimation of the clinical symptoms and therefore to possibly unnecessary neurosurgical treatments or transport of a patient. Thus, a specialized neurological examination is essential in the acute period of ICH and cannot be replaced by studying imaging scans alone. Telemedicine approaches including both clinical (mainly live stream communication) and radiological assessment by experts have been shown to be remarkably efficient and safe in assessing ischemic stroke including application of thrombolysis (19). Purely teleradiological networks for ICH evaluation by trained experts would be insufficient for immediate decision making.

Non-significant differences occur when estimating NIHSS and GCS scores in patients with hypertensive ICH versus lobar ICH. This finding is in line with a study showing a lower case fatality of patients with lobar ICH (1). This difference may at least in part be explained by the different location of lobar ICH, partly associated with less damage of eloquent brain structures and a lower rate of IVH and hydrocephalus due to the greater distance to the ventricles (20). The involvement of motor fibers and brain structures relevant for consciousness that both heavily weigh on NIHSS and GCS scores may also be more easily evaluated in the case of deep ICH than the more diverse effects of more superficial lesions. Patients with CAA-related ICH may develop some type of protective mechanism shifting away essential functions from the regions prone to bleed, for instance by previous microbleedings. Thus, subsequent lobar hemorrhage may be associated with fewer clinical symptoms than in patients with non-CAA related ICH due to ongoing neuroplasticity, similar to patients with a hemodynamically relevant stenosis of a cerebral vessel who suffer an ischemic stroke in the same region of the brain and are in part protected by ongoing collateral vessel formation (21).

To our knowledge, this is the first study addressing the reverse accuracy of clinical assessment based on cCT scans. One possible limitation of our study is that the included patients had a medium to high GCS score (GCS 7 to 15). Therefore, the question arises whether the results also fit patients with a low GCS score. In addition, we only included patients presenting to our neurological emergency unit that differs from emergency units including neurosurgery. Thus, our study possibly favored patients with a higher GCS score. However, differentiated neurological examination is not possible in most patients with a lower GCS score because such patients are often intubated before arrival in the hospital. The decision on patient care thus relies even more on other parameters such as neuroimaging. A further limitation is that statistical power is low due to small sample size. However, each participant examined 50 cCT scans with completely more than 1,000 images for this study. Neurologists are more familiar with NIHSS, neurosurgeons with GCS. This emphasizes the need for a common language or rather score for both, neurologists and neurosurgeons, for joint treatment decision making. A further concern that could be discussed is the experience of neurologists/neurosurgeons in reading cCT scans. All the same in telemedicine it is frequently up to them rather than to neuroradiologists to decide on patient care.

Our study suggests, that radiological criteria and only basic patient information cannot replace a thorough neurological examination of patients with acute ICH. Telemedical stroke networks should use their capacity for direct patient examination in adjunct to neuroimaging in order to avoid erroneous patient management and therapy.

## Data availability statement

The raw data supporting the conclusions of this manuscript will be made available by the authors, without undue reservation, to any qualified researcher.

## Author contributions

AW, K-MS, and FS contributed conception and design of the study and analysis and interpretation of the data, AW and FS wrote the first draft of the manuscript, FZ contributed analysis and interpretation of the data and wrote parts of the manuscript, SI, AS, TK, DB, and RB wrote sections of the manuscript. All authors contributed to manuscript revision, read and approved the submitted version.

### Conflict of interest statement

The authors declare that the research was conducted in the absence of any commercial or financial relationships that could be construed as a potential conflict of interest.
